# Supplementary material for: A Critical View of the Application of the APEX Software (Aqueous Photochemistry of Environmentally-Occurring Xenobiotics) to Predict Photoreaction Kinetics in Surface Freshwaters
Source: Molecules. 2019 Dec 18;25(1):9. doi: 10.3390/molecules25010009 (PMC7017383; doi:10.3390/molecules25010009)
Supplement: Supplementary file 1 [file molecules-25-00009-s001.zip › 3.2.4_gcc-4.4.0.zip!/3.2.4_gcc-4.4.0/3.2.4_gcc-4.4.0/doc/octave/html/interpreter/The-DESCRIPTION-File.html]

The DESCRIPTION File - Untitled


Next: The INDEX file,
Up: Creating Packages


---

#### 35.4.1 The DESCRIPTION File

The `DESCRIPTION` file contains various information about the
package, such as its name, author, and version. This file has a very
simple format

- Lines starting with # are comments.- Lines starting with a blank character are continuations from the
    previous line.- Everything else is of the form `NameOfOption: ValueOfOption`.

The following is a simple example of a `DESCRIPTION` file

```
     Name: The name of my package
     Version: 1.0.0
     Date: 2007-18-04
     Author: The name (and possibly email) of the package author.
     Maintainer: The name (and possibly email) of the current
      package maintainer.
     Title: The title of the package
     Description: A short description of the package.  If this
      description gets too long for one line it can continue
      on the next by adding a space to the beginning of the
      following lines.
     License: GPL version 3 or later
```

The package manager currently recognizes the following keywords

`Name`: Name of the package. `Version`: Version of the package. `Date`: Date of last update. `Author`: Original author of the package. `Maintainer`: Maintainer of the package. `Title`: A one line description of the package. `Description`: A one paragraph description of the package. `Categories`: Optional keyword describing the package (if no `INDEX` file is given this is mandatory). `Problems`: Optional list of known problems. `Url`: Optional list of homepages related to the package. `Autoload`: Optional field that sets the default loading behavior for the package. If set to `yes`, `true` or `on`, then Octave will automatically load the package when starting. Otherwise the package must be manually loaded with the pkg load command. This default behavior can be overridden when the package is installed. `Depends`: A list of other Octave packages that this package depends on. This can include dependencies on particular versions, with a format ``` Depends: package (>= 1.0.0) ``` Possible operators are `<`, `<=`, `==`, `>=` or `>`. If the part of the dependency in `()` is missing, any version of the package is acceptable. Multiple dependencies can be defined either as a comma separated list or on separate `Depends` lines. `License`: An optional short description of the used license (e.g., GPL version 3 or newer). This is optional since the file `COPYING` is mandatory. `SystemRequirements`: These are the external install dependencies of the package and are not checked by the package manager. This is here as a hint to the distribution packager. They follow the same conventions as the `Depends` keyword. `BuildRequires`: These are the external build dependencies of the package and are not checked by the package manager. This is here as a hint to the distribution packager. They follow the same conventions as the `Depends` keyword. Note that in general, packaging systems such as `rpm` or `deb` and autoprobe the install dependencies from the build dependencies, and therefore the often a `BuildRequires` dependency removes the need for a `SystemRequirements` dependency.

The developer is free to add additional arguments to the
`DESCRIPTION` file for their own purposes. One further detail to
aid the packager is that the `SystemRequirements` and
`BuildRequires` keywords can have a distribution dependent section,
and the automatic build process will use these. An example of the
format of this is

```
     BuildRequires: libtermcap-devel [Mandriva] libtermcap2-devel
```

where the first package name will be used as a default and if the
RPMs are built on a Mandriva distribution, then the second package
name will be used instead.
